# Pollination and Floral Biology of a Rare Morning Glory Species Endemic to Thailand, *Argyreia siamensis*

**DOI:** 10.3390/plants10112402

**Published:** 2021-11-07

**Authors:** Awapa Jirabanjongjit, Paweena Traiperm, Tomoki Sando, Alyssa B. Stewart

**Affiliations:** 1 Department of Plant Science, Faculty of Science, Mahidol University, Bangkok 10400, Thailand; awapa.jia@student.mahidol.ac.th (A.J.); paweena.tra@mahidol.edu (P.T.); 2 Thai-Asahi Co., Ltd., Nakhon Ratchasima 30190, Thailand; t.sando.thaiasahi@gmail.com

**Keywords:** breeding system, Convolvulaceae, histochemistry, floral nectary, pollinators

## Abstract

*Argyreia siamensis* is extremely rare, and very little is known about its reproduction. The species has colorful flowers that seem likely to attract pollinators, but population sizes are typically small (<30 individuals). To determine whether poor reproduction contributes to its rarity, we investigated its mating system and potential pollinators in two populations. We also examined the staminal trichomes and floral nectary to investigate their role in pollinator attraction. The mating system was assessed with a bagging experiment and pollinator visits were recorded with action cameras. Additionally, we tested the staminal trichomes and floral nectary for terpenes and flavonoids and examined floral nectary micromorphology via scanning electron microscope and compound light microscope. Our results reveal that *A. siamensis* is self-incompatible and dependent on pollinators; the western population was pollinated by bees (Meliponini and *Amegilla*), while the eastern population was mainly pollinated by skipper butterflies (Hesperiidae). Both staminal trichomes and the floral nectary appear to contribute to pollinator attraction through the presence of terpenes and flavonoids (in both secretory structures) and nectariferous tissue and nectarostomata (in the nectary). Our results indicate that *A. siamensis* has reliable and effective pollinators and that insufficient pollination is likely not a primary cause of its rarity.

## 1. Introduction

Tropical regions are important biodiversity hotspots [1,2], yet they are also the regions where we are still missing the most information [3]. Joppa et al. [3] estimated that 21% of flowering plants are still undescribed, and these “missing” species are most likely to be tropical species with small geographic ranges. Additionally, tropical species are more likely to be classified as Data Deficient in the IUCN Red List. For example, 17.1% of species in South and Southeast Asia are listed as Data Deficient (4,890 out of 28,629 species), while only 6.3% of species in North America are listed as Data Deficient (734 out of 11,787 species) (IUCN Red List, data retrieved 8 August 2021). Moreover, numerous studies have cautioned that Data Deficient taxa are often classified as such because they are not common, which means that many of them may actually be threatened [4,5,6,7].

One such species with a small geographic range, few known populations, and only limited information is *Argyreia siamensis* (Craib) Staples (Convolvulaceae), a rare morning glory species that is endemic to Thailand [8,9]. *Argyreia siamensis* has tubular–funnelform flowers that have a white corolla tube and bright purple corolla lobes. Flowering occurs from July to November, and fertilized flowers develop into brown berries with red sepals [10]. This species was originally known to occur only in the northern and western parts of Thailand, although a population was also recently discovered in an eastern province. While this species was initially described in 1911 [11], we still know very little about its ecology and causes of rarity. The species produces large, colorful flowers that seem likely to attract pollinators, but population sizes are small (typically less than 30 individuals) and no nectar is observed within flowers (Awapa Jirabanjongjit, personal observation), raising the question of whether insufficient pollination is one factor contributing to its rarity. The objective of this study was, therefore, to study the plant-pollinator interactions of *A. siamensis*, examining a population in western Thailand and a population in eastern Thailand. Specifically, we examined the mating system (via a pollination experiment), potential pollinators (by observing floral visitors), and floral attractants and rewards (using anatomical and histochemical techniques). Such information is important for assessing the conservation status of *A. siamensis*, which is currently labeled as data deficient. 

## 2. Results

### 2.1. Mating System

**Figure 1:**
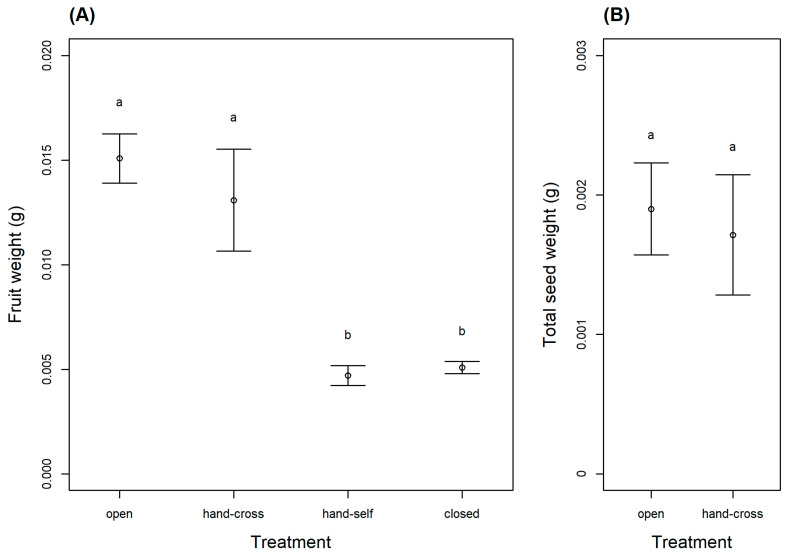
Results of the pollination experiment conducted on *Argyreia siamensis* in the western population (Ratchaburi, Thailand) showing (**A**) fruit weight and (**B**) total seed weight for each experimental treatment (open, hand cross-pollinated, hand self-pollinated, and closed) (mean ± SE). The hand self-pollinated and closed treatments never produced seeds, so these treatments are absent from the graph on the right. Within each graph, treatments with different letters are significantly different (*p* < 0.05).

**Figure 2:**
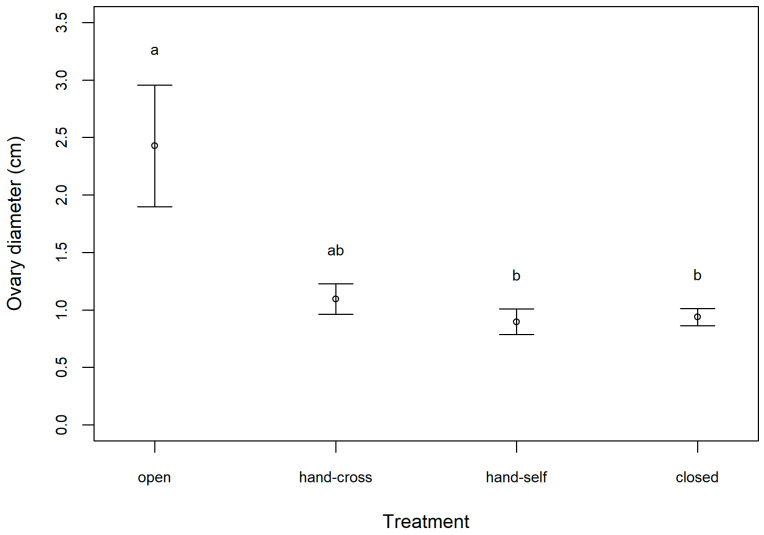
Results of the pollination experiment conducted on *Argyreia siamensis* in the eastern population (Nakhon Ratchasima, Thailand) showing ovary diameter for each experimental treatment (open, hand cross-pollinated, hand self-pollinated, and closed) (mean ± SE). Treatments with different letters are marginally significantly different (*p* < 0.10).

In the western population, fruit and seed sets were found only in the open (natural) and hand cross-pollinated treatments. LMM results revealed that treatment significantly influenced fruit weight (${Χ}_{3}^{2}=44.7$, *p* < 0.001; Figure 1A). Tukey’s post hoc further revealed that the open and hand cross-pollinated treatments produced significantly heavier fruits than the closed and hand self-pollinated treatments (*p* < 0.001; Figure 1A). As the close and hand self-pollinated treatments did not produce seeds, only open and hand cross-pollinated treatments could be compared for seed weight, and there was no significant difference between the two treatments (${Χ}_{1}^{2}=0.31$, *p* = 0.58; Figure 1B). When examining ovary diameter in the eastern population, LMM results revealed significant differences between treatments overall (${Χ}_{3}^{2}=11.5$, *p* = 0.01), but the post hoc test revealed only marginally significant differences between the open and hand self-pollinated treatments and the open and closed treatments (*p* < 0.1; Figure 2). 

### 2.2. Pollinator Observations

**Figure 3:**
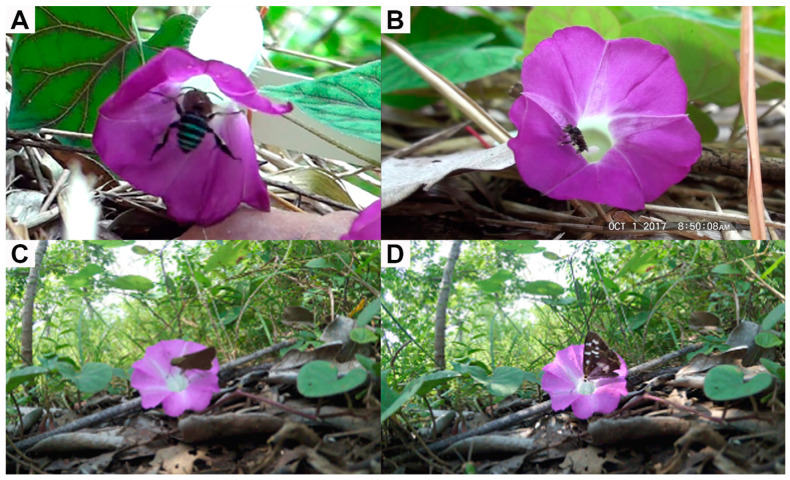
Common pollinators of *Argyreia siamensis*: (**A**) *Amegilla* sp., (**B**) Meliponini sp., (**C**) *Pelopidas* sp., and (**D**) *Udaspes folus*.

**Figure 4:**
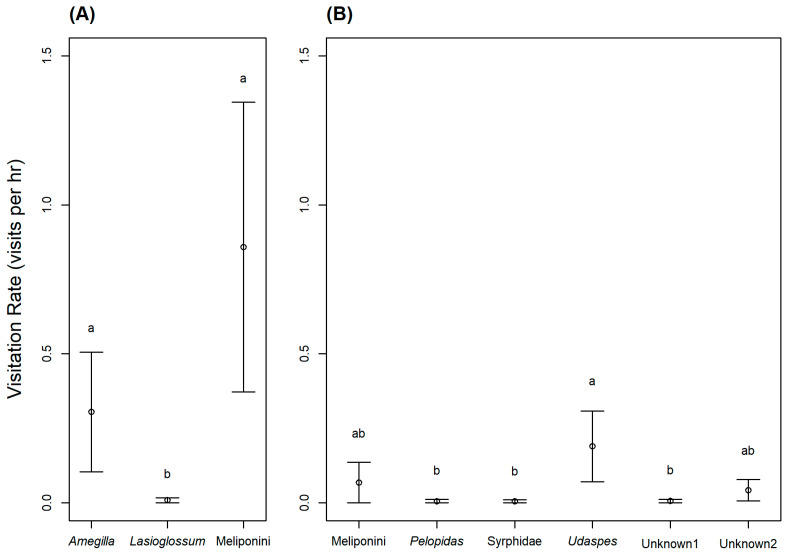
Pollinator visitation rates (mean ± SE) at *Argyreia siamensis* flowers in (**A**) the western study population (Ratchaburi, Thailand) and (**B**) the eastern study population (Nakhon Ratchasima, Thailand). Within each graph, the visitation rates of pollinator taxa with different letters are significantly different (*p* < 0.05).

In the western population, the only observed animal visitors were blue-banded bees (*Amegilla* sp.; Figure 3A), sweat bees (*Lasioglossum* sp.), and stingless bees (tribe Meliponini; Figure 3B). Based on their behavior, all three taxa were potential pollinators. Meliponini primarily visited between 06.00 and 08.00 h, and then visits declined. They typically landed on the stigmas or stamens while foraging for pollen, and sometimes crawled inside the flower for nectar. *Amegilla* bees mostly visited between 07.00 and 14.00 h. *Amegilla* bees are substantially larger than Meliponini bees (Figure 3), and when they crawled into the corolla tube to forage on nectar, their thorax visibly contacted the stigmas and stamens, and pollen was visible along the dorsal thorax. We also observed a *Lasioglossum* sweat bee visit once, and it traveled along the stigmas and anthers in the process of searching for nectar. The visitation rates of these three taxa were significantly different (${Χ}_{2}^{2}=14.88,$ *p* < 0.001; Figure 4A). *Lasioglossum* sweat bees visited flowers significantly less often than *Amegilla* blue-banded bees (*p* = 0.016), and Meliponini stingless bees (*p* = 0.004), but the visitation rates of *Amegilla* and Meliponini bees were not significantly different (*p* = 0.755) (Figure 4A). 

In the eastern population, we observed seven diurnal and three nocturnal animal taxa visit the flowers. Four taxa (nocturnal slugs and snails, and two diurnal ant taxa) are likely not true pollinators. The slugs and snails foraged on the flowers (including the corolla and some of the floral reproductive parts), while two of the ant taxa never contacted the stigmas or anthers. Among the animals that likely contribute to the pollination of *A. siamensis* (i.e., contacted stigmas and anthers), we observed two species of skipper butterflies (*Pelopidas* sp. and *Udaspes folus*; Figure 3C,D), syrphid flies (Syrphidae), stingless bees (Meliponini), and two ant taxa (one diurnal and one nocturnal). However, four of these potential pollinator taxa (*Pelopidas* sp., Meliponini, Syrphidae sp., and unknown ant 1) were only observed visiting flowers once, and one taxon (unknown ant 2) was only observed twice. The most frequent pollinator of *A. siamensis* in the eastern population was *U. folus*, which would land on the corolla and insert their proboscis into the corolla tube to collect nectar, contacting floral reproductive parts in the process. Visitation rates of these six taxa were significantly different (${Χ}_{5}^{2}=13.31,$ *p* = 0.02). *Udaspes* butterflies visited flowers significantly more often than *Pelopidas* butterflies (*p* = 0.048), syrphid flies (*p* = 0.045), and unknown ant sp. 1 (*p* = 0.048) but not Meliponini stingless bees (*p* = 0.10), or unknown ant sp. 2 (*p* = 0.25) (Figure 4B). 

### 2.3. Histochemistry

**Figure 5:**
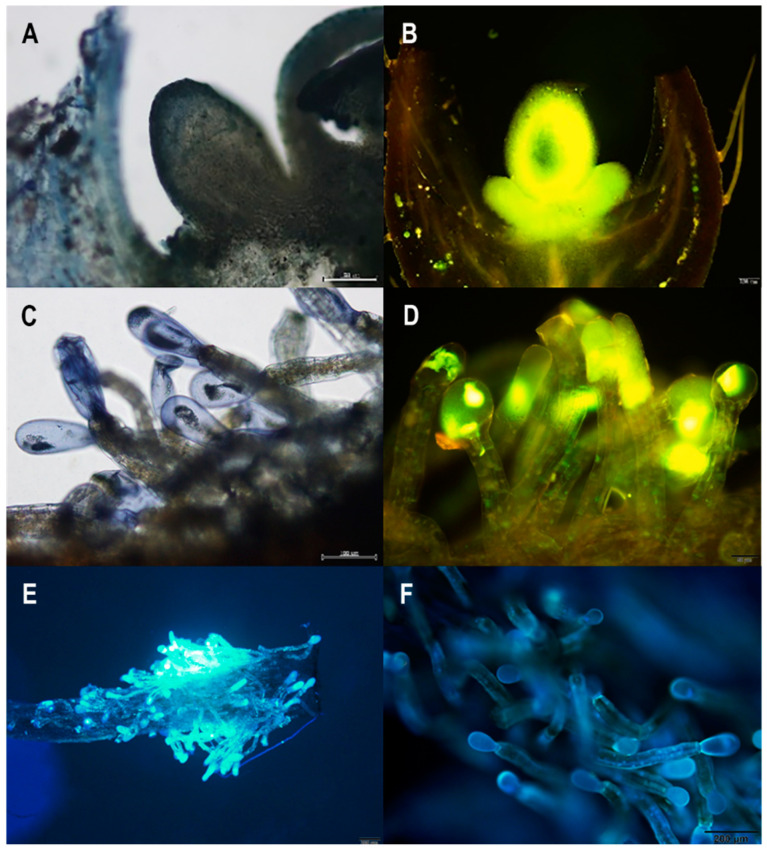
Histochemical results of (**A**,**B**) the floral nectary and (**C**–**F**) staminal trichomes of *Argyreia siamensis*. The floral nectary was positive for both (**A**) terpenes, stained with NADI reagent, and (**B**) flavonoids, stained with Naturstoff reagent A. Similarly, the staminal trichomes were positive for both (**C**) terpenes, stained with NADI reagent, and (**D**) flavonoids, stained with Naturstoff reagent A. Moreover, (**E**,**F**) normal, unstained staminal trichomes viewed under a UV filter demonstrated natural fluorescence.

We detected terpenes and flavonoids in both the floral nectary (Figure 5A,B) and the staminal trichomes (Figure 5C,D). Our investigation of autofluorescence under ultraviolet wavelengths revealed that the floral nectary did not fluoresce, but the staminal trichomes did, especially at the head of the trichomes (Figure 5E,F).

### 2.4. Floral Nectary Anatomy and Micromorphology

**Figure 6:**
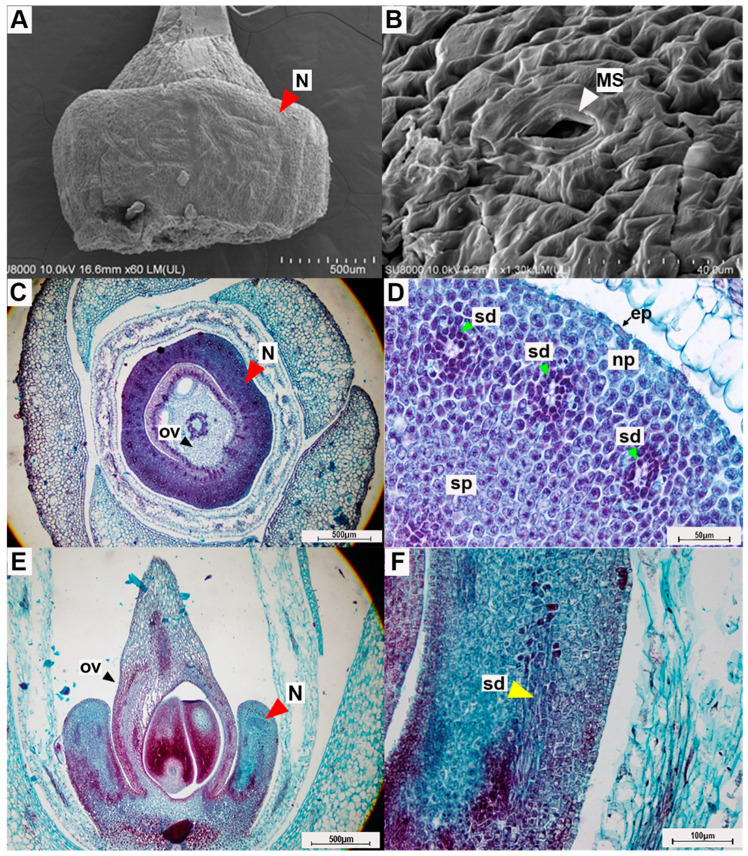
Photographs from micromorphological investigation of the floral nectary of *Argyreia siamensis*: (**A**) the entire floral nectary (which surrounds the ovary) and (**B**) surface of the floral nectary viewed under a scanning electron microscope; (**C**) transverse section of a mature flower showing the nectary and (**D**) transverse section showing close-up details of the nectary tissue; (**E**) longitudinal section of the flower and (**F**) longitudinal section of the floral nectary showing a close-up view of the secretory duct. In photos C-F, sections were stained with safranin-O and fast green. Abbreviations: N = nectary, MS = modified-stomata, ep = epidermis, ov = ovary, np = nectariferous parenchyma, sp = subnectariferous parenchyma, sd = secretory duct. Scale bars: (**A**) 500 µm; (**B**) 40 µm; (**C**) 500 µm; (**D**) 50 µm; (**E**) 500 µm; (**F**) 100 µm.

The floral nectary of *A. siamensis* is a very pale yellow, has a discoidal shape, and forms a ring surrounding the lower portion of the superior ovary at the base of the receptacle (Figure 6A,C,E). The scanning electron microscope revealed stomata scattered across the apical surface of the floral nectary (Figure 6A,B). Anatomically, the transverse (Figure 6C,D) and longitudinal (Figure 6E,F) sections of the floral nectary revealed the epidermis and ground tissue, which was composed of two regions, the nectariferous parenchyma, and subnectariferous parenchyma. The epidermis was arranged in a single-cell layer that was oriented periclinally ridged, and cuticle and trichomes were absent. Below the epidermis, nectariferous parenchyma cells were larger than the epidermis cells, and they consisted mainly of oval cells that were loosely organized. Within the nectariferous parenchyma were secretory ducts. The subnectariferous parenchyma cells were located below the nectariferous parenchyma and were similar in size to the nectariferous parenchyma cells but had less dense cytoplasm. The subnectariferous parenchyma cells were oval-shaped and exhibited packed orientation.

## 3. Discussion

### 3.1. Mating System

The pollination experiments revealed that *A. siamensis* is self-incompatible, as both the closed and hand self-pollination treatments did not set fruit or seed. This finding is consistent with previous studies reporting self-incompatibility in Convolvulaceae species (i.e., *Ipomoea wolcottiana* [12], *I. bahiensis* [13], *I. trifida* [14], *I. pes-caprae* [15]), although the family appears to have diverse mating systems, as other studies have reported self-compatible species (i.e., *I. hederacea* var. *integriuscula* [16]; *I. nil*, *Merremia aegyptia*, and *Jacquemontia evolvuloides* [13]; *I. carnea* subsp. *fistulosa* [17]), and species with mixed mating systems (i.e., *I. habeliana* [18], *Calystegia* [19], *M. macrocalyx* [20]). Self-incompatibility is an important mechanism that promotes cross-pollination [21,22]. However, self-incompatible species are highly dependent on pollinators, and require reliable and effective pollinators for pollen transfer [23], as insufficient or ineffective visitation can lead to pollen limitation [24,25,26]. 

Our pollination experiments further revealed that *A. siamensis* does not suffer from pollen limitation in our study populations, as our measures of pollination success (fruit weight, seed weight, and ovary diameter) did not differ significantly between the open and hand cross-pollination treatments. Pollen limitation is generally more common in self-incompatible species than self-compatible species [19,27], and previous studies have observed pollen limitation in self-incompatible morning glory species [19,28,29,30]. Various causes for pollen limitation have been reported, including inadequate pollinator visits (*Merremia palmeri* [28]), small population sizes (*Calystegia collina* [29]), and poor quality pollen loads (*Ipomoea hederacea* and *I. indica* [30]). Moreover, pollen limitation may occur when self-incompatible plants experience fertilization limitation [31,32], as was reported for *C. hederacea* and *C. japonica*, which experienced fertilization limitation due to an insufficient number of compatible pollen grains [19]. In contrast to the above studies, pollen limitation was not observed in the self-incompatible *Ipomoea asarifolia* [33], similar to our findings for *A. siamensis*. Since the floral visitors of *A. siamensis* appear to be reliable and effective pollinators, insufficient fruit and seed set are likely not a primary cause of the limited distribution of this endemic species.

### 3.2. Pollinator Observations

When considering both foraging behavior and visitation rates, the likely pollinators of *A. siamensis* are stingless bees (Meliponini), blue-banded bees (*Amegilla*), and skipper butterflies (particularly *U. folus*). These results are consistent with previous research, as many species in the morning glory family are pollinated by bees and butterflies [12,13,17,30,33,34,35,36,37,38,39,40,41,42,43]. Stingless bees are often abundant in tropical pollinator assemblages [44,45,46], including in Thailand [47,48,49], and many tropical species of morning glory are known to be pollinated by stingless bees, such as *Ipomoea carica*, *I. grandifolia*, and *I. nil* [36]; *I. aquatica* [43]; *I. wolcottiana* [12]; *I. hieronymi* [50]; *Merremia aegyptia* [33]; *M. macrocalyx* [20]; and *M. dissecta* var *edentada* [37]. Reports of blue-banded bees visiting Convolvulaceae species are less common, but they have been observed visiting *I. digitata* [51], *Jaquemontia* sp. [52], and *I. aquatica* [43]. Moreover, Kato et al. [51] reported that, in Laos, *Amegilla* bees typically pollinate perennial, tubular flowers, which corresponds with the habit and morphology of our study species. Butterflies are also known to visit the flowers of Convolvulaceae species; however, they appear to be less important pollinators than bees due to their less reliable and infrequent visits [17,40,53]. Our results also indicate that skipper butterflies are less frequent visitors than bees, but they appear to be important pollinators in areas where bee visitation is low, as in the case we observed in the eastern population.

Interestingly, the composition and visitation rates of potential pollinators differed between our two study populations. In the western population, we observed only bees, whereas in the eastern population, we observed mostly butterflies, and occasionally bees, ants, and flies. Stingless bees were the only pollinator taxa observed visiting *A. siamensis* in both populations, although they visited flowers in the western population much more frequently than in the eastern population. The observed pollinator differences between the two populations may be due to the different habitats. In the western site, our study species was found on the forest floor of a bamboo forest bordering a waterfall, which might explain the abundance of bee visits at this site. Heard [45] reported that stingless bees often nest in stems or branches, including the stems of bamboo. Additionally, blue-banded bees build their nests in the soil, and Sandeep and Muthuraman [54] observed *Amegilla zonata* building nests in a bamboo garden. In contrast, the eastern population was located in grassland on high ground surrounded by hills (175–271 m above sea level). The more open habitat in the eastern site may explain the presence of butterflies and syrphid flies, which were not observed in the forested western population. Previous studies have reported that many butterfly species prefer open habitats over forested habitats [55,56,57], especially smaller butterflies [58] such as the Hesperiidae butterflies observed in this study. Similarly, Hall and Reboud [59] found that syrphid flies were more common in open habitats than wooded habitats. Our study suggests that *A. siamensis* likely depends on both bee and butterfly pollinators, depending on habitat type and the pollinator community present.

### 3.3. Histochemistry

We observed terpenes in both the floral nectary and staminal trichomes of *A. siamensis*. Terpenes have many functions in plants, including attracting pollinators and seed dispersers [24,60,61,62], as they are volatile compounds that can be found in flowers and fruits [63] and can even accumulate in nectar [64]. Terpenes emitted from floral tissues, in particular, are often specific to pollinator attraction [65]. Moreover, several studies have demonstrated that terpenes are perceived by insects [62,66]. For example, studying antenna response has demonstrated that many pollinators can detect terpenes, including hawkmoths [66,67], honeybees [68,69], and stingless bees [70,71]. Another study showed that terpenes are emitted from the petals, floral nectaries, stigmas, and anthers of *Arabidopsis* flowers, which function as both a defensive mechanism against pathogens and also as short-distance attractants for insect pollinators [72]. Since our study species is self-incompatible and dependent on pollinators for reproduction, the presence of terpenes in the floral nectary and glands of staminal trichomes suggests that *A. siamensis* produces these volatile compounds to attract insect pollinators.

We also observed flavonoids in the floral nectary and staminal trichomes. Flavonoids are secondary metabolites commonly found in flowering plants that contribute to floral color and have various biological functions, including pollinator attraction [73,74,75,76,77,78]. While many flavonoids are well-known for their role in pigmentation, such as anthocyanins [79], others are colorless to the human eye, such as flavones and flavonols [79,80]. These “colorless” flavonoids can actually absorb UV wavelengths and produce UV patterns, which can act as nectar guides for bees [74,77,80]. In this study, observation of *A. siamensis* flowers under a fluorescence microscope with a UV filter revealed that the staminal trichomes, although not the floral nectary, fluoresced under UV wavelengths. Since the main pollinators of *A. siamensis* are bees, which forage more efficiently with nectar guides [81,82], we hypothesize that flavonoids in the staminal trichomes may help guide bee pollinators to the floral nectary. Flavonoids have also been found in nectar [64], which may explain why we also detected them in the floral nectary, although their function is still unclear [64].

### 3.4. Floral Nectary Anatomy and Micromorphology

Microscopic observation of the floral nectary of *A. siamensis* revealed that the structure is similar to that of other species in the family Convolvulaceae [50,83,84], and provides insight into its function. Similar to other morning glory species, *A. siamensis* has a discoidal nectary that surrounds the base of the ovary. Galetto and Bernardello [50] reported similar descriptions of floral nectaries for six *Ipomoea* (Convolvulaceae) species and suggested that it may be a conserved character within the family. The floral nectary is derived from nectariferous tissue, which comprises the epidermis, nectary parenchyma, and subnectary parenchyma [85,86,87,88], as we also observed in *A. siamensis*. The epidermis and nectary parenchyma contribute directly to nectar production and secretion, while the subnectary parenchyma appears to contribute indirectly to nectar production [88,89]. We also observed stomata distributed across the surface of the floral nectary, similar to previous reports for *Ipomoea* [50], *Anemopaegma album* [90], *Viburnum opulus* [91], *Oenothera* [92], and *Geranium macrorrhizum* and *G. phaeum* [93]. Galetto and Bernardello [50] described three types of stomata distribution along the nectary surface: uniform, only on the apex and base of the nectary, and only on the apex of the nectar; apical distribution was the most common type in their study, and also what we observed for *A. siamensis*. The stomata observed on floral nectaries are actually modified stomata that have lost the ability to close [88]; such stomata have been referred to as nectarostomata [88], and they serve to secrete nectar that is produced in the nectary parenchyma [94]. Both bees [95] and butterflies [96,97] forage on nectar, and our findings provide additional support for the importance of the floral nectary in attracting pollinators to *A. siamensis* flowers.

### 3.5. Potential Causes of Rarity in A. siamensis

Our results indicate that the rarity of *A. siamensis* is not caused by insufficient pollination. Instead, the small population sizes and limited distribution of *A. siamensis* may be due to low germination rates, poor dispersal, negative impacts caused by human activity, or some combination of these factors. It is likely that *A. siamensis* has low germination rates given that seedlings have never been observed at our study sites (Awapa Jirabanjongjit and Tomoki Sando, personal observation). We hypothesize that seed predators consume ripe fruits, as we typically only observe young fruits and empty bracts, indicating where a fruit had been removed (Tomoki Sando, personal observation). As Burmese striped squirrels (*Tamiops mcclellandii*) are often observed in the eastern study site (Tomoki Sando, personal observation), and rodents are known seed predators [98] (Crawley 2000), it is possible that this species is a seed predator of *A. siamensis*. Low germination rates (due to seed predation and/or poor germination ability) may thus explain why populations typically have fewer than 30 individuals, although empirical data are needed to confirm this conjecture. Additionally, poor dispersal ability may contribute to the limited distribution of *A. siamensis*. While the manner of seed dispersal has not been reported for *A. siamensis*, previous studies have reported other members in the genus *Argyreia* to be zoochorous (although the specific animal disperser taxa were not specified; [99,100,101]. Further observation is needed to determine whether *A. siamensis* has any seed dispersers. Finally, *A. siamensis* populations may be negatively impacted by habitat loss and other anthropogenic activity. For example, the western population occurs in an area that was mined for granite ore for nearly four decades before the government restored the area, and it is currently a popular eco-tourism site due to scenic waterfalls and views (Awapa Jirabanjongjit, personal communication). On the other hand, the eastern population appears to benefit from the moderate grazing activity of domestic water buffalo and cattle. These grazers maintain open habitat that *A. siamensis* appears to prefer, and it is often found growing near cattle trails (Tomoki Sando, personal observation). Unfortunately, the lack of data on *A. siamensis* prevents us from knowing whether anthropogenic activity has reduced the distribution of this species, or if it has always had a limited distribution. Regardless, the results of a meta-analysis indicate that self-incompatible and rare species, such as *A. siamensis*, are more likely to be negatively impacted by habitat fragmentation [102], emphasizing the need to protect this rare species.

## 4. Material and Methods

### 4.1. Study Species and Study Sites

*Argyreia siamensis* (Craib) Staples belongs to the morning glory family, also known as Convolvulaceae. Initially, this species was placed in the genus *Ipomoea* [11] until its fruits were found and proved that this species belongs to *Argyreia* [8,9]. According to the Flora of Thailand, *A. siamensis* is perennially deciduous with annual rhizomes [10]. Its leaves are ovate or ovate-orbicular and have pale leaf veins that are sometimes outlined with purple. Flowers have five stamens and a tubular–funnelform corolla; the corolla tube is white, and the corolla lobes are bright purple. When visually examining flowers, no nectar was observed (Awapa Jirabanjongjit, personal observation). *Argyreia siamensis* has brown berries with red sepals. This endemic species was originally thought to be found only in the northern and western regions of Thailand but has recently been found in the eastern region as well. It generally grows in the shaded understory of dipterocarp forests or in grassy areas on poor, rocky soil, over granite rock at altitudes of 475–1110 m. Flowering occurs from July to November [10], and flowers are open from around 05.00 h until approximately 06.00 h the following day (Awapa Jirabanjongjit, personal observation). Fruits are fully mature approximately 4–5 weeks after fertilization (Awapa Jirabanjongjit, personal observation).

This study was conducted in two populations. The first was located in a deciduous forest in Ratchaburi Province (hereafter referred to as the “western population”), with around 10 individual plants. The second population was located in an area with both deciduous forest and grassland in Nakhon Ratchasima Province (hereafter, the “eastern population”), and had approximately 30 individual plants.

### 4.2. Mating System

To examine the mating system of *A. siamensis*, we conducted a pollination experiment with the following four treatments: open pollination (flowers were unmanipulated, and all animals could visit as normal), hand cross-pollination (each flower was emasculated before anthesis and then pollinated by hand after anthesis using pollen from a different individual), hand self-pollination (each flower was pollinated by hand using autogamous pollen), and the closed treatment (mature flower buds were covered with a mesh bag that prevented visits from animals throughout the entire anthesis period). In the western population, we used 32 flowers from 8 individuals, and in the eastern population, we used 32 flowers from 5 individuals. Fruits of successfully fertilized study flowers, as well as the withered flowers of those that were not successfully fertilized, were collected 2 weeks after the start of the experiment and dried at 60 °C for 3 days. We then weighed all fruits, withered flowers, and seeds. In the eastern population, heavy rains shortly after the pollination experiment prevented the flowers from developing into mature fruits. We, therefore, measured ovary diameter as a proxy for whether or not flowers were successfully pollinated on the assumption that larger ovaries indicated they had started to develop into fruits before the heavy rains, and that smaller ovaries had likely not been successfully pollinated.

We used linear mixed modeling (LMM; function *lmer* in the R package “lme4”) to examine the effect of pollination treatment on each of our response variables (fruit weight and seed weight for the western population, and ovary diameter for the eastern population). The response variables were ln-transformed to improve the normality of the residuals. We used pollination treatment as the fixed factor and included plant ID as a random factor. We examined whether treatment was significant through nested likelihood ratio tests and, when the fixed factor was found to be significant, used Tukey’s post hoc test to compare treatment levels (function *emmeans* in package “emmeans”). These analyses were conducted in R version 4.0.2 [103].

### 4.3. Pollinator Observations

To study the potential pollinators of *A. siamensis*, all animal visitors were recorded by either a video camera (Sony CX405, Sony, New York, NY, USA) or an action camera (Xiaomi YI Z15, Xiaomi, Beijing, China) during both day (06.00–18.00 h) and night (18.00–06.00 h) for 3 days per population. We observed 13 flowers in the western population and 11 flowers in the eastern population. All footage was reviewed, and we recorded each animal visitor, its behavior, and the time of visitation. Animals were identified to the lowest taxonomic level possible using field guides [104,105]. Animals were considered visitors if they did not contact floral reproductive structures, and pollinators if they did contact the stigmas and anthers. Only pollinators were included in subsequent analyses.

We used LMM (function *lmer* in the R package “lme4”) to determine whether there were significant differences between the visitation rates of each pollinator species. Visitation rates were ln-transformed to improve the normality of the residuals. We used pollinator taxa as the fixed factor and included plant ID as a random factor. The significance of the fixed factor was examined with nested likelihood ratio tests. When the fixed factor was significant, Tukey’s post hoc test was performed to determine which pairs of pollinator taxa were significantly different (function *emmeans* in package “emmeans”). These analyses were conducted in R version 4.0.2 [103].

### 4.4. Histochemistry

We used histochemical techniques to assess the presence of phytochemical compounds of interest in the floral nectary and staminal trichomes (i.e., trichomes found on the base of the stamens). In total, 15 flowers from each population were collected. The nectary discs of fresh flowers were free-hand sectioned (both transverse and longitudinally), and stamens were removed at the base (where the filaments attached to the petals) to examine the staminal trichomes. The nectary sections and whole stamens were stained with NADI reagent (5 flowers per population) to test for terpenes [106,107] and Naturstoff reagent A (5 flowers per population) to test for flavonoids [107,108]. We also used a fluorescence microscope (Olympus BX53) with a UV filter to observe any natural fluorescence by the floral nectary and staminal trichomes (5 flowers per population).

### 4.5. Floral Nectary Anatomy and Micromorphology

To examine the characters of the floral nectary, six flowers from different individuals were collected from each population and fixed in 70% alcohol. Three of these spirit specimens were examined with a scanning electron microscope (Hitachi SU8010) [38,109] to study the surface of the floral nectary. The other three spirit specimens were examined via anatomical techniques using the paraffin method [89,110,111,112]. Briefly, the paraffin method requires all water to be removed from the spirit specimens by first using a vacuum and then dehydrating the tissue with tert-Butyl alcohol (TBA) series [89,110,111,112]. Then, the specimens are infiltrated with paraplast and embedded in the medium [89,110,111,112]. Longitudinal and transverse sections were prepared with a sliding microtome (Leica SM2000 R), stained with safranin-O and fast green, and we then examined the nectary epidermis, nectary parenchyma, and subnectary parenchyma by light microscope (Olympus BX43 with a DP21 camera set).

## 5. Conclusions

*Argyreia siamensis* is a rare plant species endemic to Thailand, but little is known about the species, and why it is rare. Our findings reveal that *A. siamensis* is self-incompatible and therefore dependent on pollinators. However, we did not find evidence for pollen limitation, which indicates that its pollinators are reliable and effective. The main pollinators of *A. siamensis* appear to be bees (Meliponini and *Amegilla*) and butterflies (Hesperiidae). Moreover, our results suggest that *A. siamensis* uses terpenes (likely as an olfactory cue) and flavonoids (possibly as a visual cue) in the floral nectary and staminal trichomes to attract and guide pollinators. The floral nectary comprises the epidermis (with nectarostomata), nectary parenchyma (with secretory ducts), and subnectary parenchyma, all of which indicate that *A. siamensis* provides pollinators with a nectar reward, despite nectar not collecting in obvious quantities within the flower. Although *A. siamensis* is rare and has a limited geographical range, the findings from this study indicate that insufficient pollination is likely not a primary cause. Further research is necessary to determine the conservation status of *A. siamensis* and factors contributing to its rarity.

## Data Availability

The data presented in this study are openly available in Mendeley Data at DOI: 10.17632/c5m8gz95p5.1.
